# Advanced ultrawide-field optical coherence tomography angiography identifies previously undetectable changes in biomechanics-related parameters in nonpathological myopic fundus

**DOI:** 10.3389/fbioe.2022.920197

**Published:** 2022-08-16

**Authors:** Weiran Zhang, Chang Li, Yibo Gong, Nianen Liu, Yunshan Cao, Zhiqing Li, Yan Zhang

**Affiliations:** ^1^ Tianjin Key Laboratory of Retinal Functions and Diseases, Tianjin Branch of National Clinical Research Center for Ocular Disease, Eye Institute and School of Optometry, Tianjin Medical University Eye Hospital, Tianjin, China; ^2^ Department of Cardiology, Gansu Provincial Hospital, Lanzhou, China

**Keywords:** myopia, optical coherence tomography angiography, vessel density, thickness, retina, choroid

## Abstract

**Purpose:** To detect previously undetectable changes in vessel density and structural thickness, the two biomechanics-related parameters reflecting hemodynamics and tensile strength, respectively, in the peripheral and central fundi of nonpathological myopic eyes with an advanced ultrawide-field optical coherence tomography angiography (OCTA) system.

**Methods:** A cross-sectional observational clinical study was carried out by recruiting 155 eyes from 79 college students aged 18–28 years. The eyes were stratified into normal, low-myopia, medium-myopia, and high-myopia groups according to diopter. A newly developed OCTA system with scanning dimensions of 24 mm × 20 mm, acquisition speed of 400 kHz, and imaging range of 6 mm was used to examine the vessel densities of superficial vascular complex (SVC), deep vascular complex (DVC), choriocapillary (ChC), and choroidal vessel (ChV) layers, as well as the thicknesses of the inner retina, outer retina, and choroid in the nonpathological myopic eyes.

**Results:** The vessel densities in ChV at the temporal, inferotemporal, inferior, and inferonasal regions in the fundus periphery were significantly reduced in myopic subjects as compared to normal controls (all *p* < 0.05). The thicknesses of the inner retinal segments in most peripheral regions of the fundus became attenuated along with myopia severity (all *p* < 0.05). The thicknesses of the outer retinal segments were diminished at the superior and supranasal regions of the peripheral fundi of myopic subjects as compared to normal controls (all *p* < 0.05). At the central macular region, the decreased vessel densities of SVC and DVC were correlated with the attenuated thicknesses of inner retinal segments, respectively (all *p* < 0.05).

**Conclusion:** As revealed for the first time by the advanced ultrawide-field OCTA system, the two biomechanics-related parameters that include the densities of the choroidal vessels and thicknesses of the inner retina segments were significantly reduced in the periphery of nonpathological myopic fundi and the reductions were associated with myopia severity. At the central macular region, the newly developed device provides consistent results with the previous findings. Therefore, it is important to use the noninvasive, ultrawide-field OCTA with high resolution for early detection of fundus changes in subjects with nonpathological high myopia.

**Clinical Trial Registration:** clinicaltrials.gov, identifier ChiCTR2100054093.

## Introduction

Myopia is a major issue of public health, with both genetic predisposition and environmental factors playing important roles in the occurrence and development of this worldwide epidemic (Baird et al., 2020). One of the pathogenic theories proposes that the blurring visual signal from the retina leads to hemodynamic changes in the choroid, hypoxia in the avascular sclera, and mechanic and structural remodeling in the sclera, thereby facilitating myopia-associated axial elongation (Wu et al., 2018). Indeed, several studies have investigated the changes in structural thickness and vascular density in and around the macula in various degrees of myopia and revealed diminished hemodynamics and reduced structural thickness in highly myopic retinas (Benavente-Pérez et al., 2010; Li et al., 2017; Liu et al., 2021). The thickness of fundus structures may indicate the tensile strength of these tissues, thereby serving as one of the biomechanics-related metrics. The link between the vascular density and biomechanics seems inexplicit, however, it has been reported that near work, one of the most prevalent risk factors of myopia elicits transient reduction in choriocapillary blood perfusion (Pan et al., 2021). Such a subtle hemodynamic change can then be detected by the cationic mechanosensors on the single-sheath capillary endothelial cells, such as Piezo 1 (Coste et al., 2010; Hill et al., 2022), which induces endothelial cell phenotypic changes those include but are not limited to angiogenesis and dysfunctions *via* YAP/TAZ and calcium signaling pathways, respectively (Harraz et al., 2022; Yang et al., 2022). These phenotypic changes subsequently lead to alterations in vascular density, exacerbating the transient blood flow deficits into persistent or even permanent ones. Therefore, the vascular density in ocular fundus results from the initial hemodynamic changes in choriocapillaris and reflects the current hemodynamics in the retina and choroid during myopia pathogenesis and progression and can thus be viewed as another biomechanics-related parameter. Furthermore, the study of the changes in vessel density and structural thickness in the retina and choroid may facilitate early detection of fundus lesions in non-pathological myopia based on which timely intervention can be implemented to slow or even halt the myopia progression. Moreover, this type of study may shed light on the pathogenesis of the highly prevalent ocular disorder from the biomechanics perspective.

Optical coherence tomography angiography (OCTA), an emerging noninvasive, noncontact imaging approach for retinal and choroidal vessels, generates images based on the differences in amplitude, phase, or both between signals acquired from multiple optical coherence tomographic (OCT) scans at identical positions (Laíns et al., 2021). OCTA has been widely used in recent years to examine changes in fundus hemodynamics under diseased conditions, such as diabetic retinopathy, central retinal vein occlusion, age-related macular degeneration, and pathological myopia (Kashani et al., 2017). The swept source OCT (SS-OCT) utilizes light sources with wavelengths (1,040–1,060 nm) longer than those that spectral-domain OCT (SD-OCT, 840 nm) uses, thereby increasing the depth of penetration and reducing the scattering from the retinal pigment epithelium. Moreover, the swept tunable lasers and the photo detector utilized by SS-OCT empower it with a higher scan speed than that SD-OCT has (Lains et al., 2021). As a result, SS-OCT can provide high-quality images of the deep retina and choroid. Since OCTA is an extension of OCT without the need of hardware modification, the angiography of SS-OCT also rapidly advances with the improvements of SS-OCT. The ultrawide-field OCTA is the SS-OCT–based imaging system of ocular fundus structure and vasculature. The ultrawide-field OCTA devices currently employed in the clinic include Xephilio OCT-S1 (Canon) (Takahashi et al., 2019), PLEX Elite™ 9000 (Carl Zeiss Meditech) (Ledesma-Gil et al., 2021), Silverstone™ (Optos) (Sodhi et al., 2021), and Triton™ DRI (Topcon) (Sun et al., 2019). MHz FDML SS-OCT system (Optores) has high scan speed; however, it has not been approved for clinical application (Tan et al., 2021).

With the availability of ultrawide-field OCTA, the biomechanics-related parameters of the fundus such as the densities of vessel layers that reflect the hemodynamics of the retina and choroid as well as the thicknesses of fundus structures that indicate the tensile strength of these tissues can be thoroughly examined. Therefore, this study seeks to, for the first time, use the newly developed ultrawide-field OCTA system, with a scan area of 24 mm × 20 mm, imaging range of 6 mm, and acquisition speed of 400 kHz, to quantitatively analyze the changes in the biomechanics-related parameters, such as vascular density and structural thickness of the fundus in the subjects with different degrees of myopia. In addition, age has been suggested to impact upon macular hemodynamics (Yu et al., 2015). Therefore, college students were selected as the subjects of this study due to their young age and a high incidence of myopia in this population.

## Methods

### Study design

This is a cross-sectional observational study composed of normal and myopic undergraduate and graduate students from the universities in Tianjin, China. This clinical study was conducted from 1 January 2022 to 1 February 2022. The study adhered to the tenets of the Declaration of Helsinki and was approved by the ethical review committee of the Tianjin Medical University Eye Hospital [2022KY(L)-01]. Written informed consent was acquired from all the subjects prior to their recruitment in this study. The study was registered at the Chinese Clinical Trial Registry (ChiCTR2100054093).

The inclusion criteria were 1) age of 18–28 years; 2) best corrected visual acuity (BCVA) ≥ 0.8; and 3) intraocular pressure ≤ 21 mmHg (1 mmHg = 0.133 kPa). The exclusion criteria were (1) BCVA < 0.8; (2) a history of systemic diseases, such as hypertension and diabetes; (3) a history of intraocular or refractive surgery; (4) intraocular pressure > 21 mmHg or normal tension glaucoma; (5) a history of ocular trauma and surgery; (6) inability to receive examinations due to poor fixation or severe refractive media opacity; (7) drug administration during past 2 weeks; and (8) severe fundus disorders, such as retinoschisis or retinal hole that impedes observation and analysis of retinal and choroidal blood flow.

The subjects in this study were divided into four groups based on spherical equivalent refraction (SER): normal group (SER ≤ ± 0.50 D), low-myopia group (−0.50 D < SER ≤ −3.00 D), medium-myopia group (−3.00 D < SER ≤ −6.00 D), and high-myopia group (−6.00 D < SER ≤ −9.00 D).

All participants attended one visit and underwent comprehensive ophthalmic examinations which included refractive error assessment converted to SER, BCVA, intraocular pressure, color fundus photography (CR-2; Canon, Tokyo, Japan), axis length (AL, SW-9000; Suoer, Tianjin, China), and OCT (RTVue XR Avanti; Optovue, Fremont, CA, United States).

### Optical coherence tomography angiography

The OCTA was performed using the newly developed SS-OCT equipment (BM-400K BMizar; TowardPi Medical Technology Co., Ltd., Beijing, China) with a laser light wavelength of 1,060 nm, an acquisition speed of 400,000 A-scans/second, a bandwidth of 100 nm, and the axial and transversal resolutions of 3.8 µM and 10 µM in tissues, respectively. Moreover, the ultrahigh frequency real-time eye movement tracking function (128 Hz) avoids the eye movement–incurred imaging errors and the subsequent procedures of motion correction. Each identical position is scanned four times. The laser power on eye tissues is 1.88 mW, meeting the national standard (GB 7247.1–2012). It is notable that the scanning dimensions of this new machine are 24 mm × 20 mm, being 13.3-fold greater than those of the previous machine (6 mm × 6 mm). The ultrawide-field OCTA images generated by this new equipment can provide more comprehensive information on the biomechanics-related parameters such as vessel density and structure thickness of human fundus. Moreover, the imaging range of the new OCTA machine, considering the influence of the refractive index of ocular tissues, reaches as far as 6 mm. Consequently, it can monitor the hemodynamic changes of the vessel layers at different depths of the fundus. These vessel layers include the superficial vascular complex (SVC) that ranges from the inner limiting membrane (ILM) to 9 µM above the inner plexiform layer (IPL), the deep vascular complex (DVC) that spans from 9 µM above the IPL to 9 µM beneath the outer plexiform layer (OPL), the choriocapillary layer (ChC) that is from the Bruch’s membrane to 29 µM beneath the membrane, and the choroidal vessel layer (ChV) that starts from 29 µM below the Bruch’s membrane to the sclera.

**FIGURE 1:**
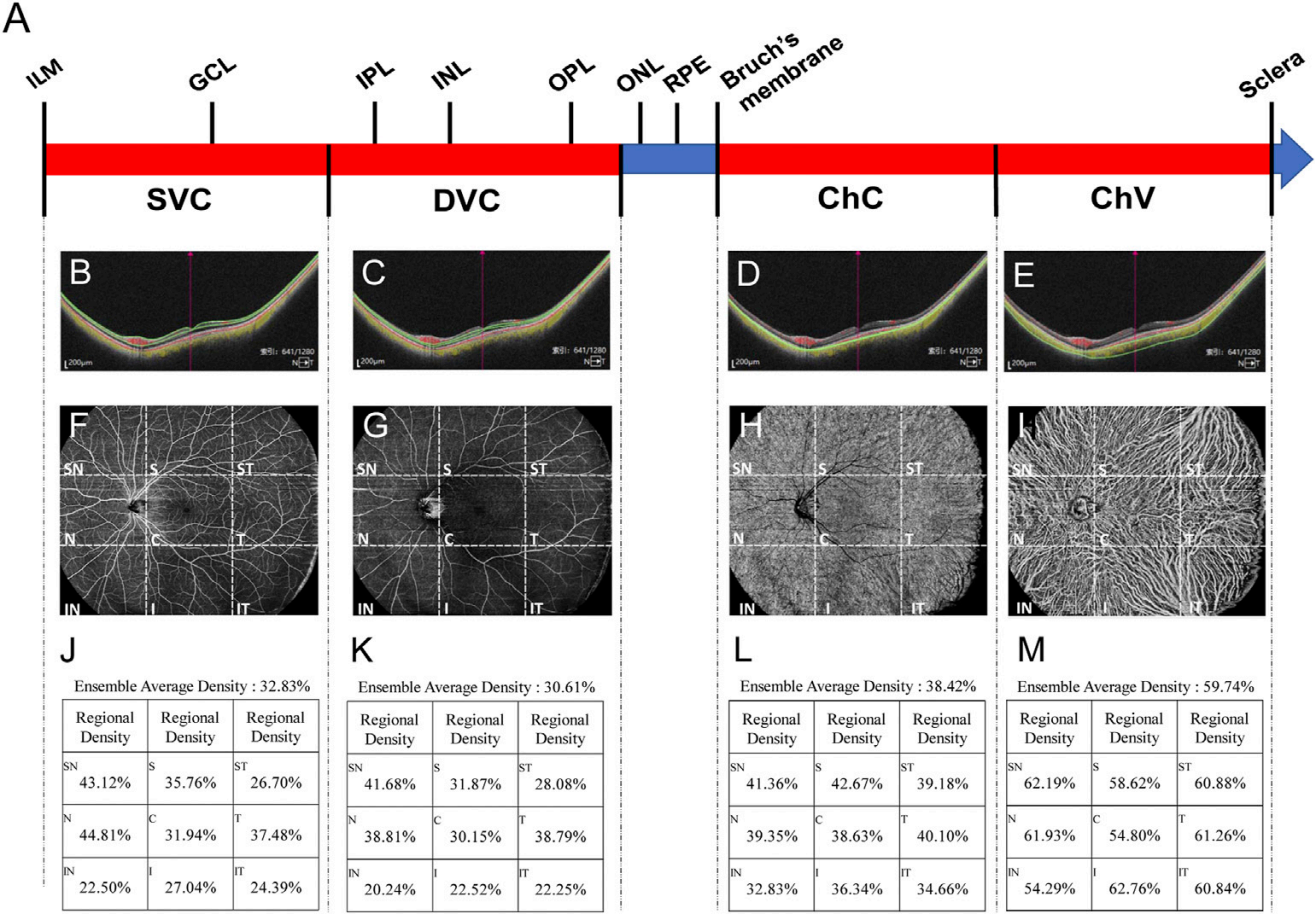
Each cross-sectional image obtained through OCTA scanning was automatically sectioned by the built-in software into four vessel layers, namely, SVC, DVC, ChC, and ChV **(A)**. In the representative image, the fovea is at the center (long red arrow), to which on the left is the nasal side and the right is the temporal side. Green lines indicate the boundaries of SVC **(B)**, DVC **(C)**, ChC **(D)**, and ChV **(E)**. Red dots represent the blood flow signals of the retina and choroidal capillary; yellow dots stand for those of choroidal vessels **(B–E)**. Both red and yellow signals were obtained through processing the scanning data with complicated algorithms. The representative of the scanned images at the enface interface is also shown with **(F) (G) (H)**, and **(I)** corresponding to SVC, DVC, ChC, and ChV vessel layers, respectively. Each image is divided by the built-in software into nine regions, namely, ST, S, SN, T, C, N, IT, I, and IN regions. Vessel density of each region at each vessel layer is quantified **(J–M)**. SVC, superficial vascular complex; DVC, deep vascular complex; ChC, choriocapillary layer; ChV, choroidal vessel layer; ST, supratemporal; S, superior; SN, supranasal; T, temporal; C, central macular; N, nasal; IT, inferotemporal; I, inferior; and IN, inferonasal.

After scanning through the ultrawide-field OCTA system, the software generated 1,280 cross-sectional images (B-scans) and this is an optimal parameter determined by the comprehensive evaluations of the scanning range, resolution, and scanning time. The whole OCTA scanning process took less than 15 s. Then, the vessel density and thickness of the retina and choroid in these images were analyzed by a built-in angiography analysis software using the algorithm of higher-order moments amplitude-decorrelation angiography (Yang and Wang, 2021). Specifically, the boundaries of SVC, DVC, ChC, and ChV vessel layers in each cross-sectional image were demarked by the software. Then, at the enface interface, all the fundus images were divided by the software into nine regions: supratemporal (ST), superior (S), supranasal (SN), temporal (T), central macular area (C), nasal (N), inferotemporal (IT), inferior (I), and inferonasal (IN) regions (Figures 1F–I). The vessel density is defined as the percentage of the vascularized area in the overall area of the montaged image at certain vascular layer in one of the nine fundus regions (Figures 1J–M). Regarding the thickness of the fundus structure, the fundus is divided into four segments: (1) the segment from the ILM to IPL, (2) the segment from the IPL to OPL, (3) the segment from the OPL to Bruch’s membrane, and (4) the segment from the Bruch’s membrane to sclera, which are essentially nourished by the abovementioned four vessel layers, respectively.

The impact of AL on ocular magnification was corrected based on the rationale put forward by Littmann (1982) (t = p × q × s; t: actual fundus dimension; p: the magnification factor of the imaging system; q: the magnification factor for the individual eye; and s: the value obtained from the imaging device). Specifically, a formula [t = (Al - 4) × s/20] was developed to correct ocular magnification for the ultrawide-field OCTA system used in this study.

Six additional myopic eyes meeting the inclusion criteria were recruited, and the OCTA scan was performed twice on each eye to measure the vessel density and structure thickness in the fundus. Intra-class correlation coefficients (ICCs) between the two measurements of individual eyes were calculated to evaluate the repeatability of the employed ultrawide-field OCTA system in this study.

### Statistical analyses

Statistical analyses were performed using the Statistical Program for Social Sciences 20.0 (IBM SPSS Inc., New York, NY, United States). The gender distribution in each group was expressed as the proportion of females to males, the differences in gender distribution among the groups were compared using the χ^2^ test. All continuous data were examined by the D’Agostino and Pearson omnibus normality test, after which they were examined by the Levene test to determine the homogeneity of variance. The data with Gaussian distribution and homogeneity of variance were expressed as mean ± SD, and the differences among the groups were analyzed using one-way ANOVA followed by the Bonferroni *post hoc* test for pairwise comparisons. The data with Gaussian distribution but uneven variance or those with non-Gaussian distribution were expressed as the median (interquartile range, 25%–75%), and the differences among the groups were analyzed using the Kruskal–Wallis test. Adjustments for multiple comparisons were conducted with Bonferroni correction. *p* < 0.05 was deemed as statistically significant.

## Results

### Demographic information

**FIGURE 2:**
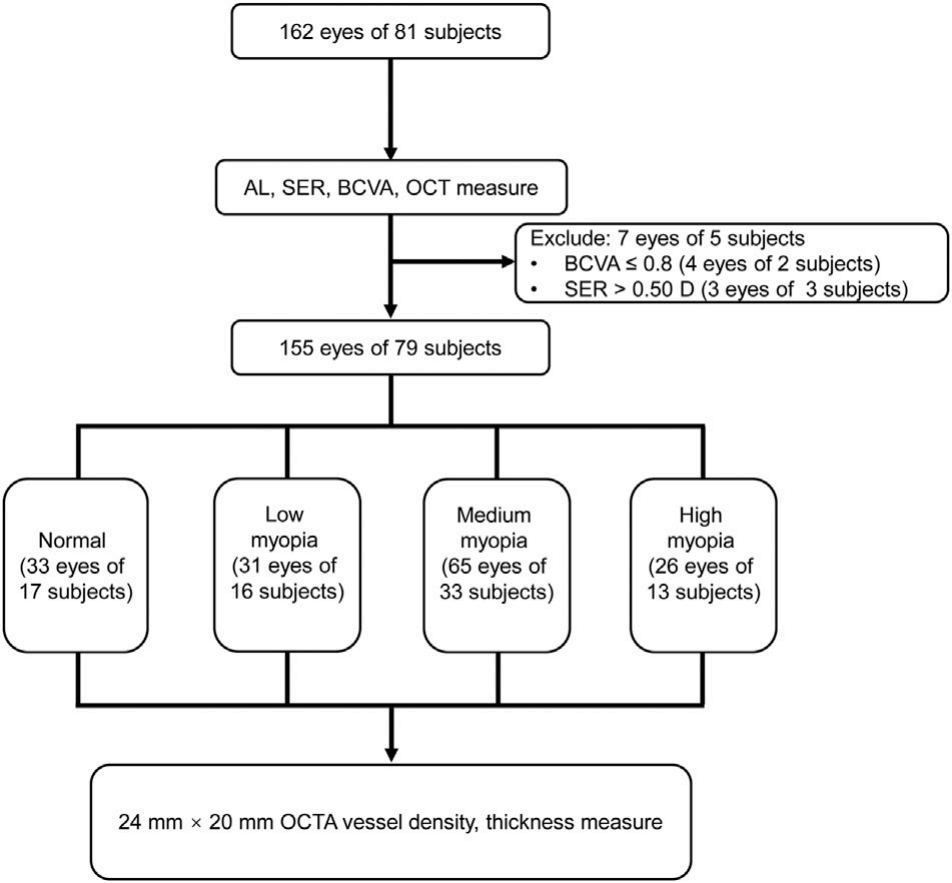
Flowchart for the current study. AL, axis length; SER, spherical equivalent refraction; BCVA, best-corrected visual acuity; OCT, optical coherence tomography; and OCTA, optical coherence tomography angiography.

A total of 162 eyes (81 subjects) were initially recruited, and 7 eyes (2 subjects) were excluded from the study according to the exclusion criteria. The remaining 155 eyes (79 subjects, 34 males, and 45 females) were stratified into normal, low-, medium-, and high-myopia groups according to SER. The normal group comprised of 33 eyes with emmetropia. The low-, medium-, and high-myopia groups had 31, 65, and 26 eyes, respectively. Each eye was then subjected to an OCTA examination with dimensions of 24 mm × 20 mm (Figure 2). Neither age nor gender distribution exhibited significant difference among the groups (*p* = 0.576 for age; *p* = 0.904 for gender distribution, Table 1). However, the AL prolonged significantly as myopia progressed; the longer the AL, the greater the severity of myopia (*p* < 0.001) and the SER was also greater as myopia became more severe (*p* < 0.001, Table 1).

**TABLE 1:** Information of the studied eyes.

Group	Nor (33 eyes)	Low (31 eyes)	Med (65 eyes)	High (26 eyes)	*p*
Age, mean (SD), y	23.39 (2.70)	24.26 (2.42)	23.86 (2.50)	24.35 (3.00)	0.576^a^
Sex (female : male)	20:13	16:15	36:29	15:11	0.904^b^
AL, mean (SD), mm	23.67 (0.53)	24.77 (0.79)	25.81 (0.80)	26.94 (1.07)	<0.001^a^
SER, mean (SD), D	−0.07 (0.23)	−2.03 (0.74)	−4.77 (0.99)	−7.44 (0.80)	<0.001^a^

a Nonparametric test.

b Chi-squared test.

Nor, normal group; Low, low-myopia group; Med, medium-myopia group; High, high-myopia group; SD, standard deviation; y, years; D, Diopter; AL, axis length; SER, spherical equivalent refraction.

### Vessel density and structural thickness in different fundus regions of the subjects with different degrees of myopia

**FIGURE 3:**
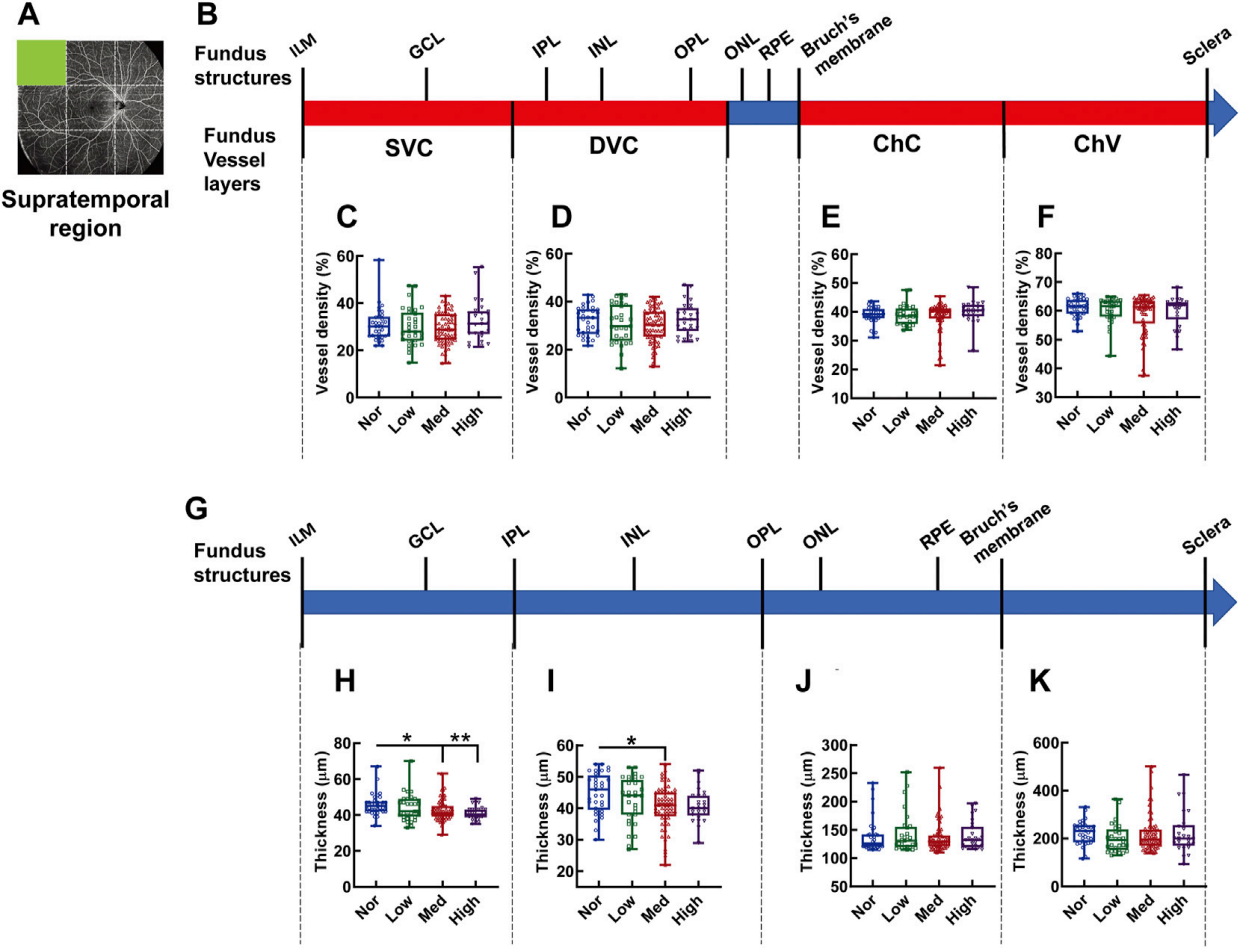
Vessel layer densities and structure thicknesses at the supratemporal region of the fundus. Position of the supratemporal region of the fundus is illustrated in **(A)**. Schematic illustration of vessel layers in the fundus is shown in **(B)**. Vessel densities of superficial vascular complex **(C)**, deep vascular complex **(D)**, choriocapillary **(E)**, and choroidal vessel **(F)** layers were quantified and compared. Schematic illustration of segments in the fundus is shown in **(G)**. Thicknesses of the segments from IML to IPL **(H)**, from IPL to OPL **(I)**, from OPL to Bruch’s membrane **(J)**, and from Bruch’s membrane to the sclera **(K)** are quantified and compared. * *p* < 0.05, ** *p* < 0.01. SVC, superficial vascular complex; DVC, deep vascular complex; ChC, choriocapillary layer; ChV, choroidal vessel layer; NOR, normal group; Low, low-myopia group; Med, medium-myopia group; High, high myopia group; IML, inner limiting membrane; IPL, inner plexiform layer; and OPL, outer plexiform layer.

In the supratemporal region, as shown by the fundus OCTA in Figures 3A,B, no difference is found in vessel densities at each layer of the fundus vessels, such as SVC (Figure 3C), DVC (Figure 3D), ChC (Figure 3E), and ChV (Figure 3F), among the groups of the subjects with different degrees of myopia. However, the thickness between ILM and IPL, the retinal segment (Figure 3G) essentially nourished by the SVC, in the supratemporal region of the normal subjects was significantly greater than that in subjects with medium (*p* < 0.05, Nor *vs.* Med, Figure 3H) and high myopia (*p* < 0.01, Nor *vs.* High, Figure 3H), but there was no difference in thickness between normal and low-myopia groups (*p* = 0.546, Nor *vs.* Low, Figure 3H). The thicknesses of other fundus segments, such as those from the IPL to OPL, from the OPL to Bruch’s membrane, and from the Bruch’s membrane to sclera, did not exhibit any differences among the groups with different degrees of myopia (Figures 3J,K), except for a small but significant difference found in the thickness from the IPL to OPL between the low and medium-myopia groups (*p* < 0.05, Low *vs.* High, Figure 3I).

**FIGURE 4:**
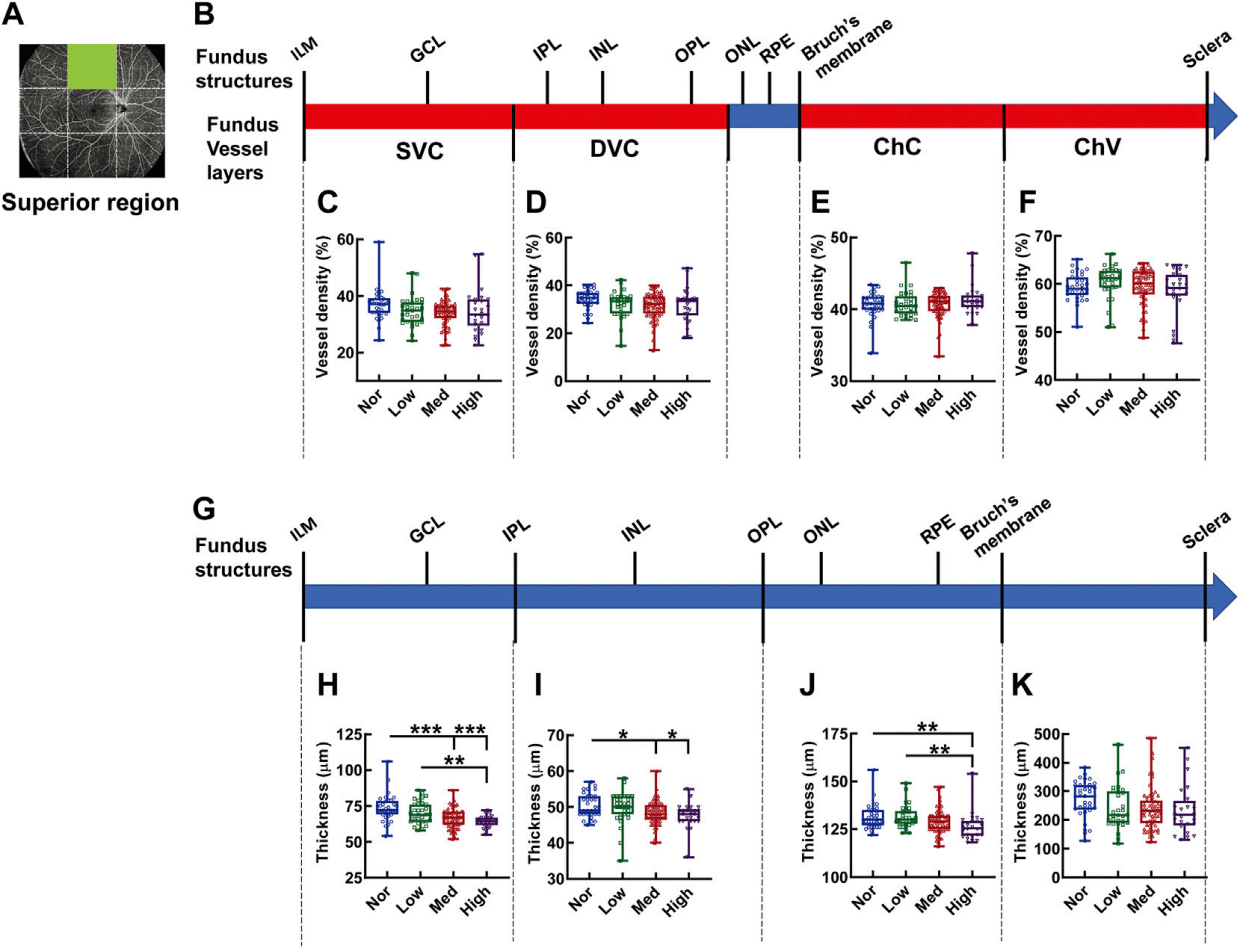
Vessel layer densities and structure thicknesses at the superior region of the fundus. Position of the superior region of the fundus is illustrated in **(A)**. Schematic illustration of vessel layers in the fundus is shown in **(B)**. Vessel densities of superficial vascular complex **(C)**, deep vascular complex **(D)**, choriocapillary **(E)**, and choroidal vessel **(F)** layers are quantified and compared. Schematic illustration of segments in the fundus is shown in **(G)**. Thicknesses of the segments from IML to IPL **(H)**, from IPL to OPL **(I)**, from OPL to Bruch’s membrane **(J)**, and from Bruch’s membrane to the sclera **(K)** are quantified and compared. * *p* < 0.05, ** *p* < 0.01, *** *p* < 0.001. SVC, superficial vascular complex; DVC, deep vascular complex; ChC, choriocapillary layer; ChV, choroidal vessel layer; NOR, normal group; Low, low-myopia group; Med, medium-myopia group; High, high myopia group; IML, inner limiting membrane; IPL, inner plexiform layer; and OPL, outer plexiform layer.

In the superior region of the fundus (Figures 4A,B), the densities in each vascular layer did not show any significant difference among the myopic groups (Figures 4C–F). By contrast, a tapering trend in the thicknesses of the fundus segments (Figure 4G) supplied by the corresponding SVC, DVC, and ChC layers at the superior region was observed among the subjects with increasingly aggravated myopia (both *p* < 0.001, Nor *vs.* Med and Nor *vs.* High, *p* < 0.01, Low *vs.* High, Figure 4H; both *p* < 0.05, Nor *vs.* Med and Nor *vs.* High, Figure 4I; *p* < 0.01, Nor *vs.* High, and Low *vs.* High, Figure 4J). However, the thickness of the choroid, which is mainly supplied by ChV, was an exception, and no significant difference was detected among the myopic groups (Figure 4K).

**FIGURE 5:**
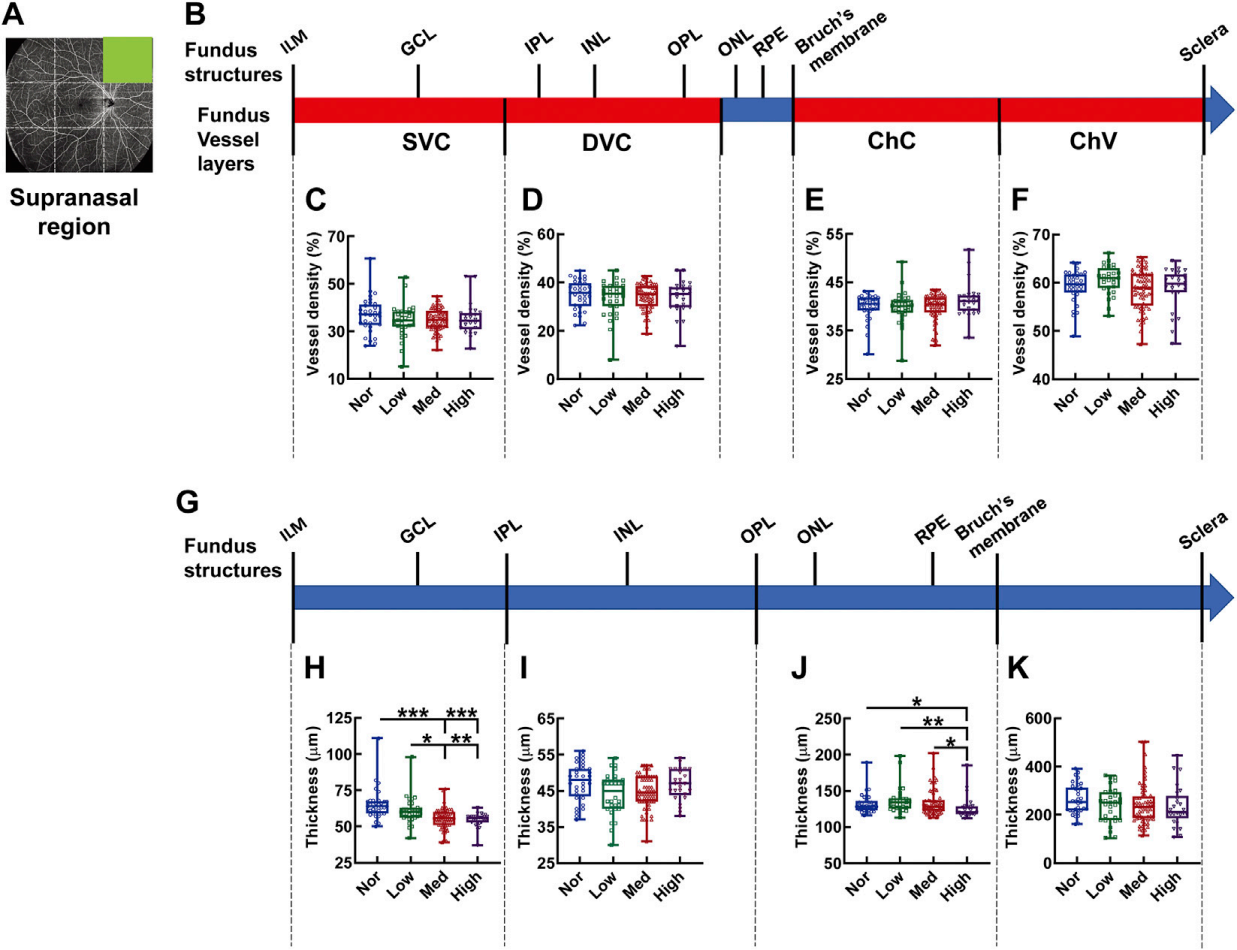
Vessel layer densities and structure thicknesses at the supranasal region of the fundus. Position of the supranasal region of the fundus is illustrated in **(A)**. Schematic illustration of vessel layers in the fundus is shown in **(B)**. Vessel densities of superficial vascular complex **(C)**, deep vascular complex **(D)**, choriocapillary **(E)**, and choroidal vessel **(F)** layers are quantified and compared. Schematic illustration of segments in the fundus is shown in **(G)**. Thicknesses of the segments from IML to IPL **(H)**, from IPL to OPL **(I)**, from OPL to Bruch’s membrane **(J)**, and from Bruch’s membrane to the sclera **(K)** are quantified and compared. * *p* < 0.05, ** *p* < 0.01, *** *p* < 0.001. SVC, superficial vascular complex; DVC, deep vascular complex; ChC, choriocapillary layer; ChV, choroidal vessel layer; NOR, normal group; Low, low-myopia group; Med, medium-myopia group; High, high myopia group; IML, inner limiting membrane; IPL, inner plexiform layer; and OPL, outer plexiform layer.

In the supranasal region of the fundus OCTA (Figures 5A,B), the densities of each vascular layer did not show any significant difference (Figures 5C–F). By contrast, there was a gradual shrinkage as myopia progressed in thickness from the IML to IPL, corresponding to the SVC-nourishing segment (*p* < 0.001, Nor *vs.* Med and Nor *vs.* High, *p* < 0.01, Low *vs.* High, *p* < 0.05, Low *vs.* Med, Figure 5H); the thickness from the OPL to Bruch’s membrane (ChC-supplying segment) of the high-myopia group was significantly attenuated as compared to those of the normal, low-myopia, and medium-myopia groups (*p* < 0.05, Nor *vs.* High, *p* < 0.01, Low *vs.* High, *p* < 0.05, Med *vs.* High, Figure 5J). The choroid thickness demonstrated no significant change among the subjects with different degrees of myopia (Figure 5K).

**FIGURE 6:**
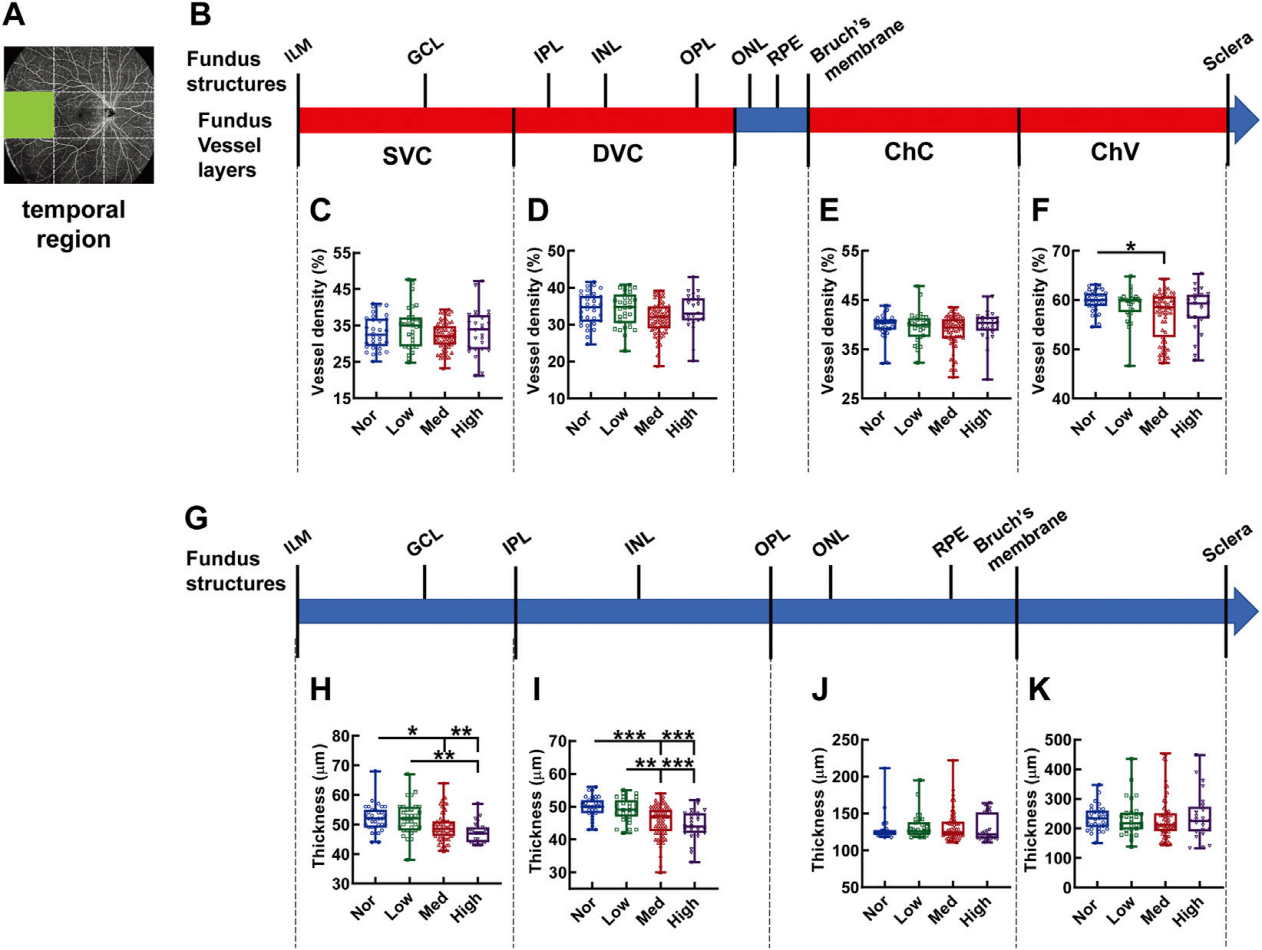
Vessel layer densities and structure thicknesses at the temporal region of the fundus. Position of the temporal region of the fundus is illustrated in **(A)**. Schematic illustration of vessel layers in the fundus is shown in **(B)**. Vessel densities of superficial vascular complex **(C)**, deep vascular complex **(D)**, choriocapillary **(E)**, and choroidal vessel **(F)** layers are quantified and compared. Schematic illustration of segments in the fundus is shown in **(G)**. Thicknesses of the segments from IML to IPL **(H)**, from IPL to OPL **(I)**, from OPL to Bruch’s membrane **(J)**, and from Bruch’s membrane to the sclera **(K)** are quantified and compared. * *p* < 0.05, ** *p* < 0.01, *** *p* < 0.001. SVC, superficial vascular complex; DVC, deep vascular complex; ChC, choriocapillary layer; ChV, choroidal vessel layer; NOR, normal group; Low, low-myopia group; Med, medium-myopia group; High, high myopia group; IML, inner limiting membrane; IPL, inner plexiform layer; and OPL, outer plexiform layer.

At the temporal region of the fundus (Figures 6A,B), the vessel density of the ChV layer in the subjects with medium myopia was reduced 31.36% as compared to that in the normal subjects (*p* < 0.01, Nor *vs.* Med, Figure 6F). No difference in vessel density was found in other vessel layers among the groups with different degrees of myopia (Figures 6C–E). Notably, a dwindling trend of thickness was observed in the segments from IML to IPL and from IPL to OPL, supplied by the SVC and DVC, respectively, along with myopia severity (*p* < 0.01, Nor *vs.* High, Low *vs.* High, *p* < 0.05, Nor *vs.* Med, Figure 6H; *p* < 0.001, Nor *vs.* Med, Nor *vs.* High, Low *vs.* High, *p* < 0.01, Low *vs.* Med, Figure 6I). The thicknesses of the other segments did not show any difference among the study groups, presumably as a result of great intragroup variabilities (Figures 6J,K).

**FIGURE 7:**
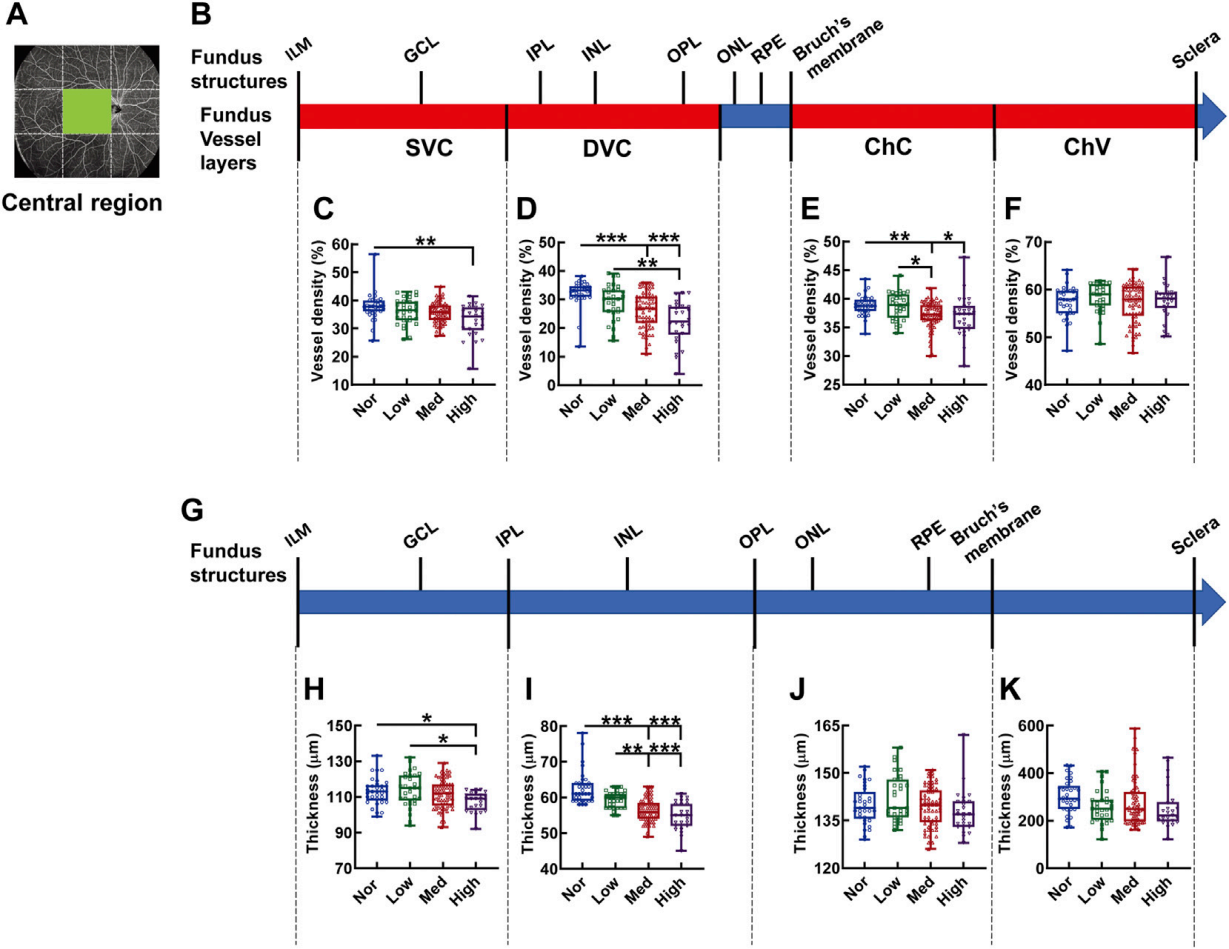
Vessel layer densities and structure thicknesses at the central macular region of the fundus. Position of the central macular region of the fundus is illustrated in **(A)**. Schematic illustration of vessel layers in the fundus is shown in **(B)**. Vessel densities of superficial vascular complex **(C)**, deep vascular complex **(D)**, choriocapillary **(E)**, and choroidal vessel **(F)** layers are quantified and compared. Schematic illustration of segments in the fundus is shown in **(G)**. Thicknesses of the segments from IML to IPL **(H)**, from IPL to OPL **(I)**, from OPL to Bruch’s membrane **(J)**, and from Bruch’s membrane to the sclera **(K)** are quantified and compared. * *p* < 0.05, ** *p* < 0.01, *** *p* < 0.001. SVC, superficial vascular complex; DVC, deep vascular complex; ChC, choriocapillary layer; ChV, choroidal vessel layer; NOR, normal group; Low, low-myopia group; Med, medium-myopia group; High, high myopia group; IML, inner limiting membrane; IPL, inner plexiform layer; and OPL, outer plexiform layer.

The central region is the only fundus region where vessel density and structure thickness displayed consistent downward trends among the subjects with myopia of different severity (Figure 7). In specifics, the vessel density of the SVC in high myopic subjects was significantly lessened when compared with that in the normal controls (*p* < 0.01, Nor *vs.* High, Figure 7C). The vessel densities of both DVC and ChC in the normal subjects were significantly higher than those in subjects with medium and high myopia (*p* < 0.001, Nor *vs.* Med, Nor *vs.* High, Figure 7D; *p* < 0.01, Nor *vs.* Med, *p* < 0.05, Nor *vs.* High, Figure 7E). Moreover, significant differences were found in the vessel densities of DVC and ChC layers even among the myopic groups (*p* < 0.01, Low *vs.* High, Figure 7D; *p* < 0.05, Low *vs.* Med, Figure 7E). The thicknesses from the IML to IPL and from the IPL to OPL paralleled the trends displayed by the densities of the SVC and DVC layers, respectively (Figures 7C,D,H,I). Furthermore, the correlation analysis revealed that the vessel densities of SVC and DVC were positively correlated to the thicknesses of the retinal segments supplied by these two vessel layers (for SVC and the segment from the IML to IPL, r = 0.229, *p* < 0.01, Supplementary Figure S1A; for DVC and the segment from the IPL to OPL, r = 0.631, *p* < 0.001, Supplementary Figure S1B), implicating that both the hemodynamics and tensile strength of the fundus tissues at the macular region contribute significantly to myopia progression. There was no significant difference in the vessel densities of ChC and ChV or in the thicknesses of the outer retina and choroid membrane (Figures 7F,K).

**FIGURE 8:**
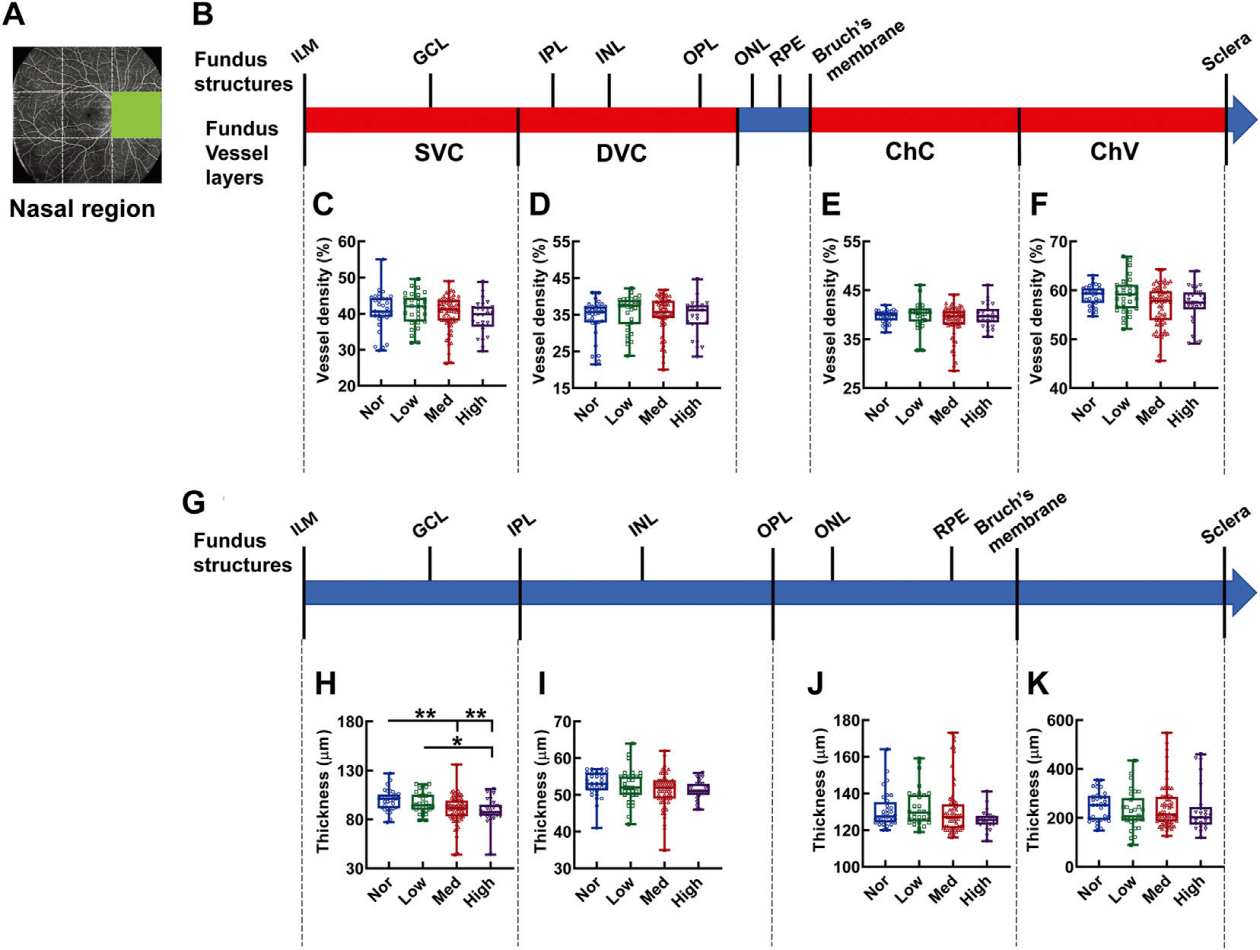
Vessel layer densities and structure thicknesses at the nasal region of the fundus. Position of the nasal region of the fundus is illustrated in **(A)**. Schematic illustration of vessel layers in the fundus is shown in **(B)**. Vessel densities of superficial vascular complex **(C)**, deep vascular complex **(D)**, choriocapillary **(E)**, and choroidal vessel **(F)** layers are quantified and compared. Schematic illustration of segments in the fundus is shown in **(G)**. Thicknesses of the segments from IML to IPL **(H)**, from IPL to OPL **(I)**, from OPL to Bruch’s membrane **(J)**, and from Bruch’s membrane to the sclera **(K)** are quantified and compared. * *p* < 0.05, ** *p* < 0.01. SVC, superficial vascular complex; DVC, deep vascular complex; ChC, choriocapillary layer; ChV, choroidal vessel layer; NOR, normal group; Low, low-myopia group; Med, medium-myopia group; High, high myopia group; IML, inner limiting membrane; IPL, inner plexiform layer; and OPL, outer plexiform layer.

In the nasal region, no difference was detected in the density of each vessel layer (Figures 8A–F). On the other hand, merely the SVC-nourishing segments in the inner retinas of the normal subjects were thicker than those of the medium and high myopic subjects (*p* < 0.01 Nor *vs.* Med, Nor *vs.* High, *p* < 0.05, Low *vs.* High, Figure 8H). The thicknesses of the other segments were not different among the groups (Figures 8I–K).

**FIGURE 9:**
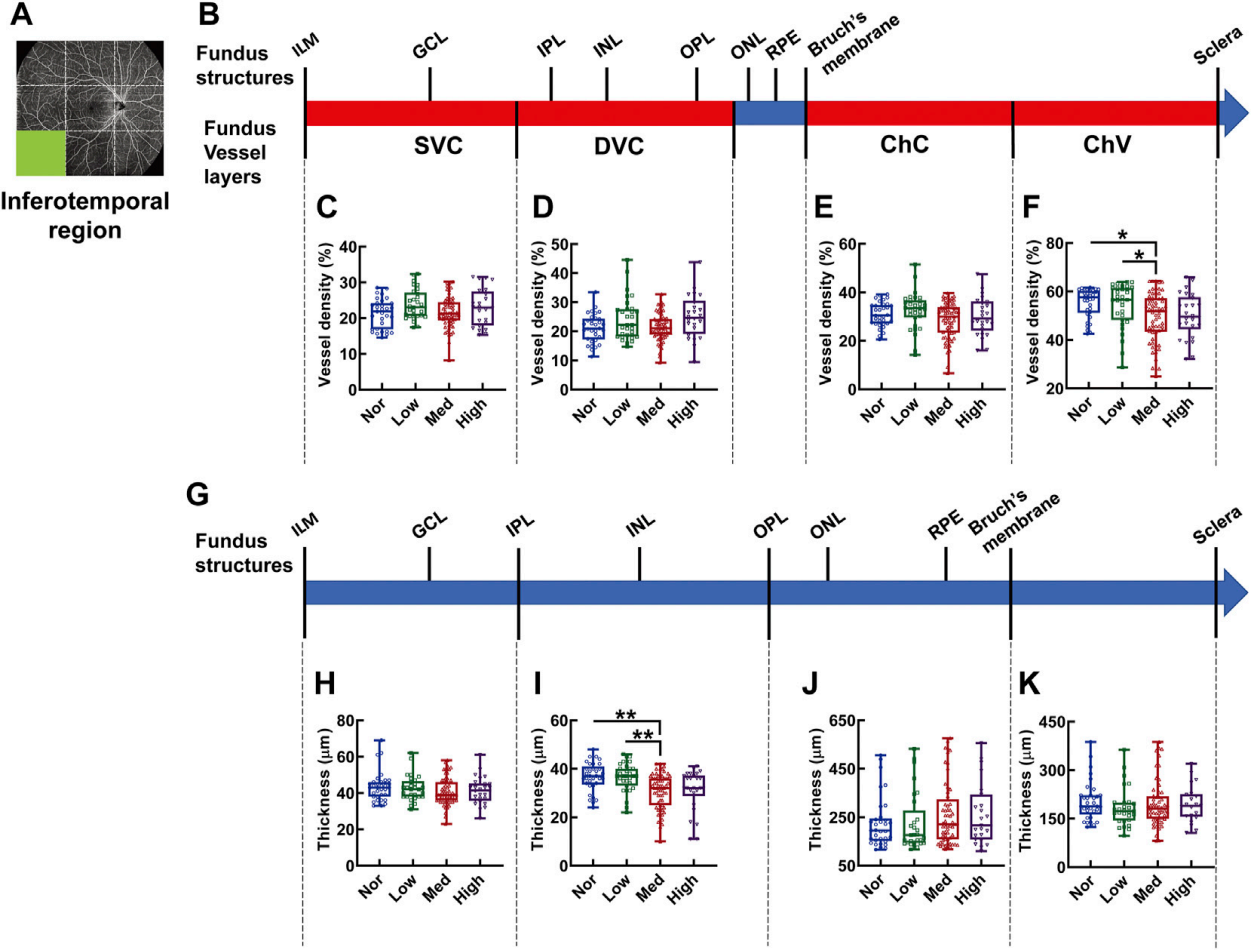
Vessel layer densities and structure thicknesses at the inferotemporal region of the fundus. Position of the inferotemporal region of the fundus is illustrated in **(A)**. Schematic illustration of vessel layers in the fundus is shown in **(B)**. Vessel densities of superficial vascular complex **(C)**, deep vascular complex **(D)**, choriocapillary **(E)**, and choroidal vessel **(F)** layers are quantified and compared. Schematic illustration of segments in the fundus is shown in **(G)**. Thicknesses of the segments from IML to IPL **(H)**, from IPL to OPL **(I)**, from OPL to Bruch’s membrane **(J)**, and from Bruch’s membrane to the sclera **(K)** are quantified and compared. * *p* < 0.05, ** *p* < 0.01. SVC, superficial vascular complex; DVC, deep vascular complex; ChC, choriocapillary layer; ChV, choroidal vessel layer; NOR, normal group; Low, low-myopia group; Med, medium-myopia group; High, high myopia group; IML, inner limiting membrane; IPL, inner plexiform layer; and OPL, outer plexiform layer.

In the inferotemporal region of the fundus, the vessel density showed significant difference in the layer of ChV and in subjects with medium myopia was 28.62% and 28.38% lower than that of normal and low myopic subjects, respectively (*p* < 0.05, Nor *vs.* Med, Low *vs.* Med, Figure 9F); whereas, the thickness from the IPL to OPL of the Med myopia group was significantly reduced in comparison to those of the normal and low-myopia groups (*p* < 0.01, Nor *vs.* Med, Low *vs.* Med Figure 9I).

**FIGURE 10:**
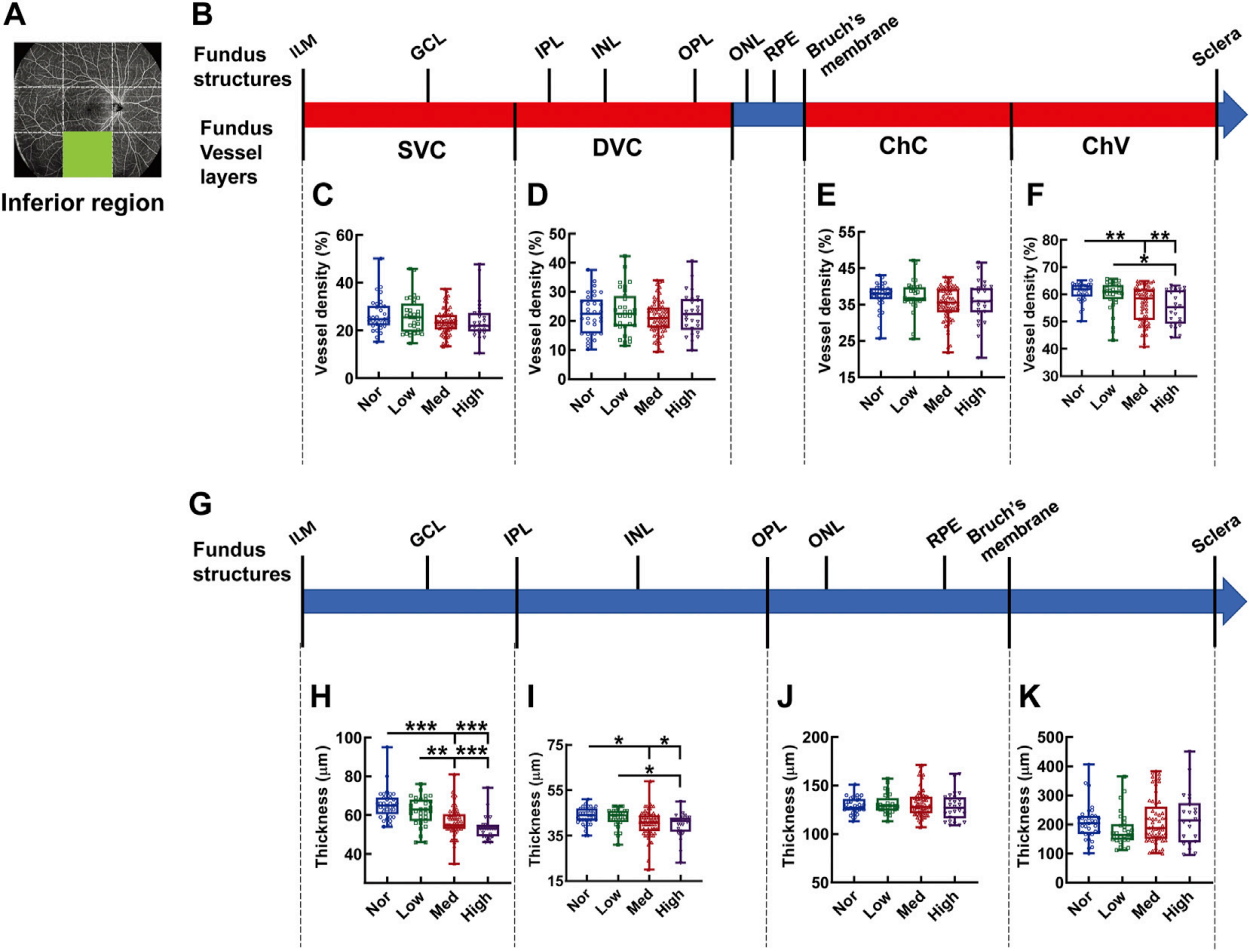
Vessel layer densities and structure thicknesses at the inferior region of the fundus. Position of the inferior region of the fundus is illustrated in **(A)**. Schematic illustration of vessel layers in the fundus is shown in **(B)**. Vessel densities of superficial vascular complex **(C)**, deep vascular complex **(D)**, choriocapillary **(E)**, and choroidal vessel **(F)** layers are quantified and compared. Schematic illustration of segments in the fundus is shown in **(G)**. Thicknesses of the segments from IML to IPL **(H)**, from IPL to OPL **(I)**, from OPL to Bruch’s membrane **(J)**, and from Bruch’s membrane to the sclera **(K)** are quantified and compared. * *p* < 0.05, ** *p* < 0.01, *** *p* < 0.001. SVC, superficial vascular complex; DVC, deep vascular complex; ChC, choriocapillary layer; ChV, choroidal vessel layer; NOR, normal group; Low, low-myopia group; Med, medium-myopia group; High, high myopia group; IML, inner limiting membrane; IPL, inner plexiform layer; and OPL, outer plexiform layer.

In the inferior region, the significant difference in vessel density was found in the layer of ChV (Figures 10A–F), where the vessel densities of the medium- and high-myopia groups were diminished in comparison to those of normal controls (*p* < 0.01, Nor *vs.* Med, Nor *vs.* High, Figure 10F) and the vessel density of the low-myopia group was significantly higher than that of the high-myopia group (*p* < 0.05, Low *vs.* High, Figure 10F). On the other hand, the thickness reduction was evident in the two inner retinal segments supplied by the SVC and DVC as myopia exacerbated (*p* < 0.001, Nor *vs.* Med, Nor *vs.* High, Low *vs.* High, *p* < 0.01, Low *vs.* Med, Figure 10H; *p* < 0.05, Nor *vs.* Med, Nor *vs.* High, Low *vs.* High. Figure 10I), while no significant change in the thicknesses of the segments supplied by the ChC and ChV was found among the groups (Figures 10J,K).

**FIGURE 11:**
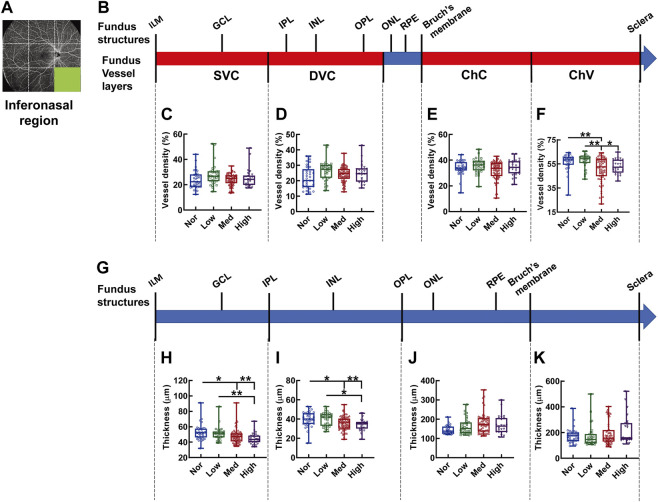
Vessel layer densities and structure thicknesses at the inferonasal region of the fundus. Position of the inferonasal region of the fundus is illustrated in **(A)**. Schematic illustration of vessel layers in the fundus is shown in **(B)**. Vessel densities of superficial vascular complex **(C)**, deep vascular complex **(D)**, choriocapillary **(E)**, and choroidal vessel **(F)** layers are quantified and compared. Schematic illustration of segments in the fundus is shown in **(G)**. Thicknesses of the segments from IML to IPL **(H)**, from IPL to OPL **(I)**, from OPL to Bruch’s membrane **(J)**, and from Bruch’s membrane to the sclera **(K)** are quantified and compared. * *p* < 0.05, ** *p* < 0.01. SVC, superficial vascular complex; DVC, deep vascular complex; ChC, choriocapillary layer; ChV, choroidal vessel layer; NOR, normal group; Low, low-myopia group; Med, medium-myopia group; High, high myopia group; IML, inner limiting membrane; IPL, inner plexiform layer; and OPL, outer plexiform layer.

In the inferonasal region, the difference in vessel density among the groups was only detected in ChV, with the vessel density in the normal controls being greater than in the medium-myopia subjects (*p* < 0.01, Nor *vs.* Med, Figure 11F) and the vessel density in the low-myopia subjects being higher than that in their medium and high counterparts (*p* < 0.01 Low *vs.* Med, *p* < 0.05, Low *vs.* High, Figure 11F). The thicknesses were significantly different in the segments of the inner retina, where stepwise decrement was observed among the groups with exacerbating myopia (*p* < 0.01, Nor *vs.* High, Low *vs.* High, *p* < 0.05, Nor *vs.* Med, Figure 11H; *p* < 0.01, Nor *vs.* High, *p* < 0.05, Nor *vs.* Med, Low *vs.* High, Figure 11I).

To assess the repeatability of the newly developed ultrawide-field OCTA system, the vascular density and structural thickness were measured twice, and additionally in six myopic eyes. According to the results described previously, the vessel densities of the ChV in the peripheral fundus and DVC in the central macula, as well as the thicknesses from ILM to IPL in the peripheral fundus and IPL to OPL in the central region exhibited the most significant differences among the experimental groups, therefore, the layer of the ChV and the segment from the IML to IPL were selected as representatives to evaluate the repeatability of the measurements in the peripheral fundus, while the DVC layer and IPL to OPL segment were selected for the macula. The results showed that the vessel densities of the ChV and the thicknesses from the IML to IPL in all fundus peripheral regions in the first measurement were highly consistent with the corresponding metrics in the second measurement, with the ICCs ranging from 0.813 to 1.000 (all *p* < 0.05, Supplementary Figures S2, S3). In the central region, the vessel density of the DVC and the thickness from the IPL to OPL displayed even better consistency between the two measurements, with the ICC being 1.000 for the former (*p* < 0.001, Supplementary Figure S4) and 0.916 for the latter (*p* = 0.003, Supplementary Figure S4). These results demonstrate good repeatability of the ultrawide-field OCTA system used in our study.

## Discussion

Myopia is becoming an epidemic around the globe, particularly in Asia. However, the currently available interventional modalities, such as glass-wearing, orthokeratology, and topical drug applications, cannot effectively prevent its incidence or halt its progression. The progression of myopia accompanies hemodynamic changes in both the central and peripheral regions of the retina and choroid. These changes were monitored by color Doppler (Shimada et al., 2004) and fluorescein angiography (Kaneko et al., 2014; Moriyama et al., 2017) more than a decade ago. However, color Doppler does not provide sufficient resolution to view delicate vasculature in the retina and choroid. Fundus fluorescein angiography (FFA) remains the gold standard to examine the structure of retinal vessels and the integrity of the blood–retina barrier, yet its invasive nature and potential cytotoxicity render it unideal for examining non-pathological myopia (Kornblau and El-Annan, 2019; Šranková et al., 2022). The emergence of OCTA, a fast and noninvasive technology with high resolution, has greatly improved the convenience, compliance of subject, and accuracy in examining fundus hemodynamics and vasculature (Hormel et al., 2021). However, the majority of on-duty OCTA systems are SD-OCT and have limitations. For example, only an image with dimensions of 3 × 3 mm or 6 × 6 mm is captured at the macular area (Nesper and Fawzi, 2018; Lal et al., 2021; Robbins et al., 2021). Moreover, the short-wavelength light (840 nm) generated by the superluminescent diode in SD-OCT merely reaches the SVC, DVC, and ChC layers in the fundus and leaves the ChV undetected due to its shallow penetration and scattering from the retinal pigment epithelium. In this study, a newly developed, SS-OCT based, ultrawide-field OCTA system was employed. The scanning image acquired from the new OCTA has the dimensions of 24 mm × 20 mm and is equivalent to 80° in the fundus, thereby covering both macula and periphery in the fundus. In addition, the 1,060-nm light source empowers a 6-mm imaging range, which is particularly suitable for observing the fundus vessels of myopic eyes whose axes have abnormally prolonged.

The OCTA system employed in our study has a high-frequency eye movement–tracking system and an automatic software system, which effectively eliminate the involuntary interference of subjects. In addition, the system built-in stringent quality control of scanned images also augments the reliability of the measured metrics. For example, the vascular density and structural thickness of the central macular region measured in our study are consistent with those obtained by SD-OCT in a previous study (Wang et al., 2021). Meanwhile, the OCTA images of the peripheral retina captured by this machine are of good quality to facilitate diagnosis of retinitis pigmentosa (Zheng et al., 2022).

In the OCTA images captured at eight peripheral regions of the fundus, the differences in one of the biomechanics-related parameters, vessel density, between the normal and myopic subjects were predominantly found in the ChV layer at the temporal, inferotemporal, inferior, and inferonasal regions (Figures 6F, 9–11). These previously undescribed findings provide direct evidence demonstrating the reduced density in the ChV layer of the peripheral fundus in young non-pathological myopic subjects, with the temporal and inferior regions being the most affected sites. By contrast, another biomechanics-related parameter, the attenuation of thickness in the fundus segments, was more readily detected. The thickness of the segment from the IML to IPL was decreased in seven peripheral regions, namely, the supratemporal, superior, supranasal, temporal, nasal, inferior, and inferonasal regions, along with myopia severity (Figures 3–6, 8H, 10, 11). Moreover, the segment from the IPL to OPL became thinner in six peripheral regions, namely, supratemporal, superior, temporal, inferotemporal, inferior, and inferonasal regions (Figures 3, 4, 6I, 9–11); the thickness of the outer retina supplied by ChC diminished in the superior and supranasal regions in myopic subjects (Figures 4, 5). These observations suggest that the myopia-induced thickness changes in the peripheral fundus favor inner retina nourished by the SVC and DVC. It is noted that in the peripheral fundus, the changes in vessel density are incongruent with those in structural thickness, implicating no causal or correlational relationship between the two in the fundus periphery under non-pathological myopia and their presumably differential contributions to myopia progression. Investigations of the molecular and cellular mechanism are warranted to rationally explain this inconsistency.

However, these previously unidentified changes in biomechanics-related parameters in the peripheral fundus should not be ignored for the following three reasons. First, during retinal development, the peripheral retina grows at a different rate than its central counterpart (Zeng et al., 2013), indicating the relative independence of the former from a physiological perspective. In addition, an experimental study on the primate model of myopia showed that infant monkeys whose peripheral retinas had been subjected to form deprivation while the central retinas were left undisturbed exhibited refractive progression and eccentric eye growth, suggesting the sufficiency of changes in the peripheral retina to the pathogenesis and progression of myopia (Smith et al., 2009). In addition, a community-based clinical study revealed that a significant portion of non-pathological myopic people bore lesions in the peripheral retina, particularly those with high myopia at a young age (Lai et al., 2008). Therefore, the changes in the periphery of non-pathological myopic fundus may herald the pathological lesions under aggravated myopia in the future, and this is particularly relevant for the subjects less than 35 years of age. Moreover, the findings in this study also highlight the importance of using the advanced ultrawide-field OCTA system for early and thorough detection of fundus changes in subjects with non-pathological high myopia.

The OCTA images of the central macular region show that both the vessel density and retinal thickness were significantly decreased as the degree of myopia advanced (Figures 7C–E), the trend of which is consistent with published reports. For instance, Wu et al. (2021) reported greater reductions of vessel density in the ChC layer at the macula in subjects with more advanced myopia. Jiang et al. (2021) observed that the vessel densities of the SVC and DVC at the macula were significantly decreased in high myopic eyes as compared to those of normal ones. Furthermore, in the current study, the reduction in the densities of SVC and DVC at the central macular region was correlated with the attenuation in thicknesses of inner retinal segments along with myopia severity (Supplementary Figure S1). These findings agree with a previous study that shows a strong correlation between the vascular perfusing parameters and retinal thickness at the macular region (Yu et al., 2016) and demonstrate that the new ultrawide-field OCTA system can provide novel information on the peripheral fundus without losing the sensitivity and resolution at the macula. The results at the macular region also suggest that a decline in the two biomechanics-related parameters such as vascular density and inner retinal thickness contributes significantly to myopia progression.

Then, an interesting question would be what causes the changes in vessel density and structure thickness in the non-pathological myopic fundi? It is generally believed that the axial extension during myopia progression stretches the posterior pole of the fundus, the area corresponding to the central macular region (Asano et al., 2019). This mechanical stretching, at least at the macula, makes the diameters of blood vessels in the retina and choroid smaller (La Spina et al., 2016) than the normal ones, thereby reducing blood perfusion, vessel density, and thickness of fundus structures. Yet, the molecular and cellular candidates in this theory require further investigations. Then, which change, the vessel density or structural thickness, comes first during myopia progression? The results from different studies are discrepant. Some have suggested that a thinner retina with lower metabolic demand and tensile strength results in blood flow reduction, indicating that the attenuation in structural thickness occurs first (Man et al., 2013). By contrast, other studies have shown that vessel density in the fundus can rapidly respond to environmental stimuli, and near work can reduce fundus blood perfusion within a short period of time, implying the decrease in vessel density as an initiator (Pan et al., 2021). Therefore, carefully designed large cohort studies are warranted to reconcile the discrepancy.

There are limitations in this study. First, this is a cross-sectional study, the dynamic changes in vessel density and structure thickness in the fundi of young people with non-pathological myopia cannot be monitored, therefore, long-term follow-up studies are warranted. Second, the sample size of this study is small, with 26 (13 subjects) to 65 (33 subjects) eyes in each group. Thus, a large sample-sized, random-controlled, double-blinded clinical study is needed to corroborate the results of this study.

Collectively, by using the advanced ultrawide-field OCTA system with scanning dimensions of 24 mm × 20 mm, imaging range of 6 mm, and acquisition speed of 400 kHz, this study provides the previously undetected evidence that the two biomechanics-related parameters such as choroidal vascular density and inner retinal thickness are reduced in the periphery of non-pathological myopic fundus. The reductions are associated with myopia severity and implicate the risk of pathological lesions. The changes of vessel density and retinal thickness at the macula are consistent with previous findings (Wang et al., 2021). The ultrawide-field OCTA system should be used for the early detection of vascular and structural changes in the fundi of non-pathological myopic eyes.

## Data Availability

The raw data supporting the conclusion of this article will be made available by the authors, without undue reservation.
