# Supplementary material for: Advanced ultrawide-field optical coherence tomography angiography identifies previously undetectable changes in biomechanics-related parameters in nonpathological myopic fundus
Source: Front Bioeng Biotechnol. 2022 Aug 16;10:920197. doi: 10.3389/fbioe.2022.920197 (PMC9424555; doi:10.3389/fbioe.2022.920197)
Supplement: Supplementary file 1 [file DataSheet1.PDF]

Supplementary figures

Supplementary figure 1

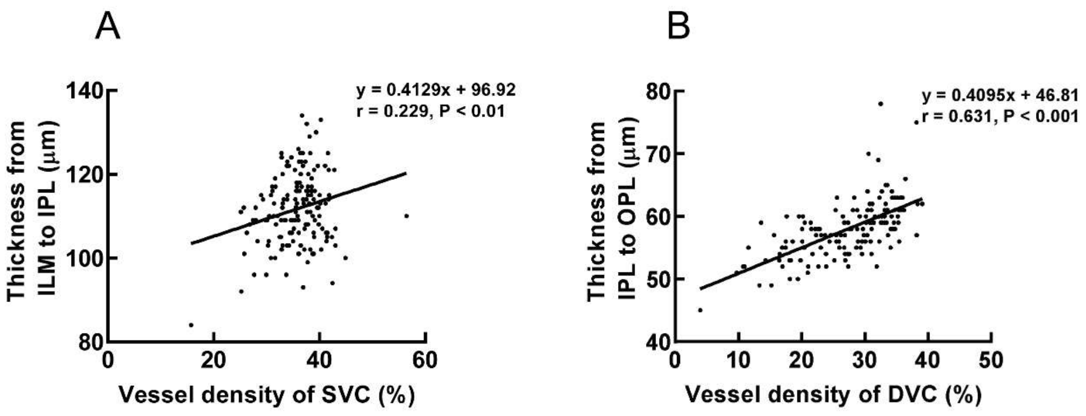

Supplementary figure 1. Correlation between structure thickness and vessel density at central macular region. (A) The thickness from IML to IPL was correlated with the density of SVC (r = 0.229, P < 0.01). (B) The thickness of from IPL to OPL was correlated with the density of DVC (r = 0.631, P < 0.001). IML = inner limiting membrane; IPL = inner plexiform layer; OPL = outer plexiform layer; SVC = superficial vascular complex; DVC = deep vascular complex.

Supplementary figure 2

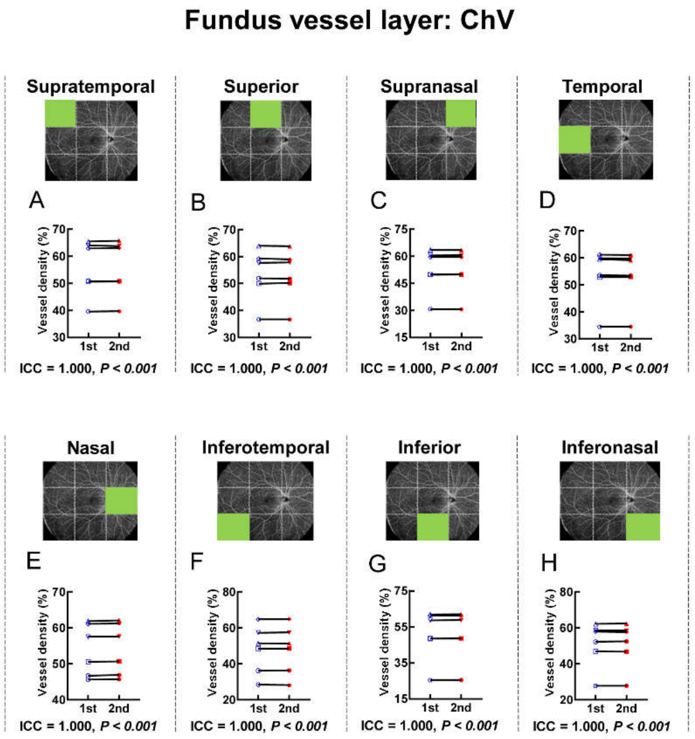

Supplementary figure 2. The repeatability in the measurement of vessel densities of ChV in

the peripheral fundus. The vessel densities of ChV in 6 myopic eyes were recorded during the 1st and 2nd measurements in the supratemporal (A), superior (B), supranasal (C), temporal (D), nasal (E), inferotemporal (F), inferior (G), and inferonasal (H) regions of the fundus. The corresponding data from the 2 measurements were compared, and the ICC and *P* values were shown. ChV = choroidal vessel layer; ICC = intraclass correlation coefficients.

Supplementary figure 3

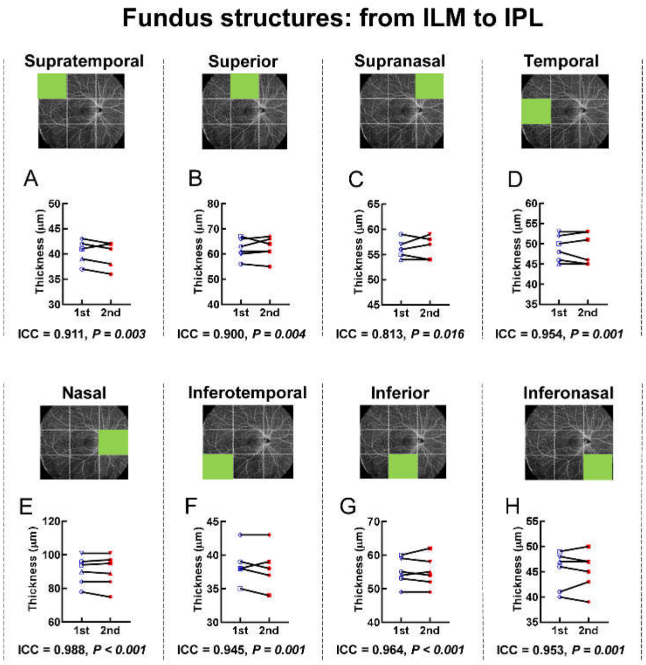

Supplementary figure 3. The repeatability in the measurement of thicknesses from ILM to IPL in the peripheral fundus. The thicknesses from ILM to IPL in 6 myopic eyes were recorded during the 1st and 2nd measurements in the supratemporal (A), superior (B), supranasal (C), temporal (D), nasal (E), inferotemporal (F), inferior (G), and inferonasal (H) regions of the fundus. The corresponding data from the 2 measurements were compared, and the ICC and *P* values were shown. ILM = inner limiting membrane; IPL = inner plexiform layer; ICC = intraclass correlation coefficients.

Supplementary figure 4

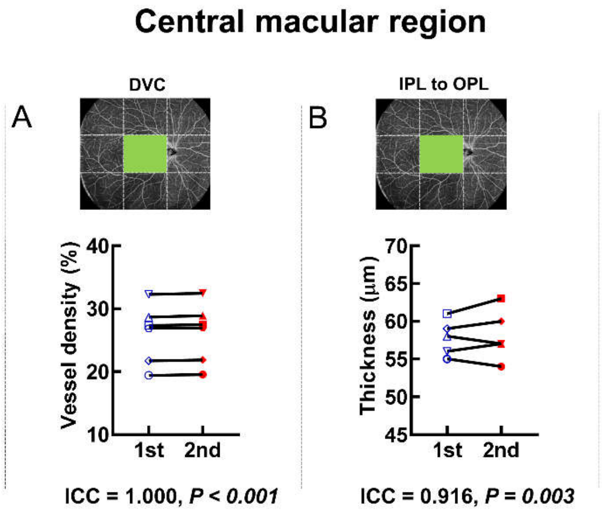

Supplementary figure 4. The repeatability in the measurement of vessel density and structural thickness in the central macula. The vessel density of DVC (A) and the thickness from IPL to OPL (B) in 6 myopic eyes were recorded during the 1st and 2nd measurements in the central macular region. The corresponding data from the 2 measurements were compared, and the ICC and *P* values were shown. DVC = deep vascular complex; IPL = inner plexiform layer; OPL = outer plexiform layer; ICC = intraclass correlation coefficients.
